# Lower variability in female students than male students at multiple timescales supports the use of sex as a biological variable in human studies

**DOI:** 10.1186/s13293-021-00375-2

**Published:** 2021-04-22

**Authors:** Benjamin L. Smarr, Annick Laure Ishami, Aaron E. Schirmer

**Affiliations:** 1 Department of Bioengineering and the Halıcıoğlu Data Science Institute, University of California San Diego, 9500 Gilman Drive Drive, La Jolla, CA 92093 USA; 2 Department of Biology, Northeastern Illinois University, 5500 N. St. Louis Ave, Chicago, IL 60625 USA

## Abstract

**Background:**

Men have been, and still are, included in more studies than women, in large part because of the lingering belief that ovulatory cycles result in women showing too much variability to be economically viable subjects. This belief has scientific and social consequences, and yet, it remains largely untested. Recent work in rodents has shown either that there is no appreciable difference in overall variability across a wealth of traits, or that in fact males may show more variability than females.

**Methods:**

We analyzed learning management system logins associated to gender records spanning 2 years from 13,777 students at Northeastern Illinois University. These data were used to assess variability in daily rhythms in a heterogeneous human population.

**Results:**

At the population level, men are more likely than women to show extreme chronotypes (very early or very late phases of activity). Men were also found to be more variable than women across and within individuals. Variance correlated negatively with academic performance, which also showed a gender difference. Whereas a complaint against using female subjects is that their variance is the driver of statistical sex differences, only 6% of the gender performance difference is potentially accounted for by variance, suggesting that variability is not the driver of sex differences here.

**Conclusions:**

Our findings do not support the idea that women are more behaviorally variable than men and may support the opposite. Our findings support including sex as a biological variable and do not support variance-based arguments for the exclusion of women as research subjects.

**Supplementary Information:**

The online version contains supplementary material available at 10.1186/s13293-021-00375-2.

## Background

Persistent beliefs that ovarian cycles make women more variable, and therefore experimental confounds, have contributed to the exclusion of women as research subjects and have resulted in males being the default sex in both human and animal experiments [1–6]. These persistent beliefs have left female subjects substantially understudied compared to men [1, 5, 7, 8]. Despite national policies to try and mitigate this exclusion [9, 10], both the belief and its negative effect remain prevalent [11–15].

Recently, a number of studies have looked at animal data—from genetics to time series analysis of physiology—and found that in fact males show either equal or slightly greater variance than females across many traits [16–18]. For example, when using continuous physiological and behavioral recordings, we previously demonstrated that male mice show more variance within a day than female mice show across an entire 4-day ovarian cycle [16].

To our knowledge, analyses directly comparing variance over time in men and women have not been reported, despite “common knowledge” to the contrary. In part, this has been due to difficulty obtaining data that is both longitudinal (following individuals over time to capture daily and monthly cycles) and wide enough to cover a large population of men and women. We found such a dataset when uncovering the impact of circadian variation on student performance, using logins to the Northeastern Illinois University (NEIU) campus learning management systems as proxies for activity [19]. Here, we use these same data to explore the differences in variance across multiple timescales in men and women, across and within individuals.

Turning the old belief into a hypothesis, we sought to confirm or reject whether data from women is more variable than data from men over the same time frame. In this case, the data contain no information about the phase of any individual’s ovarian cycles or the presence or absence of hormonal birth control that might affect cycling. Therefore, women are treated as randomized with respect to the cycle phase, hypothetically maximizing their across-individual variance, and so making for an ideal test case.

## Methods

Under the Northeastern Illinois University institutional review board (IRB) protocol #16-073 MO1, data from 13,777 students were collected, de-identified, and processed as described previously [19]. Briefly, student data contained time-stamped events for each time a student logged into NEIU’s learning management system. Login events for a specific student ID (randomized pin to assure anonymity) were identified with both demographic and academic variables. The only demographic information used here was self-reported gender, which is available in the university records as a binary (M/F). We, therefore, use the conventional terms “gender,” “men,” and “women” when referring to human subjects, and “sex,” “male,” and “female” when referring across species or to effects referenced in the literature (e.g., “sex as a biological variable,” but see [20, 21]). Academic variables include semester GPAs, courses taken, start and end times of individual courses, and individual course grades. A threshold of 12 entries per individual was applied to all entries. If an individual did not meet this threshold or was missing a gender descriptor, then that individual was excluded from these analyses. This filtering had already been carried out in the generation of the data analyzed here, so that all 13,777 individuals were included in all analyses in this manuscript.

Variables were processed in the R statistical package [22], and subsequent analyses were carried out with both R and Matlab 2019a. The date of each login event was compared against the individual student’s class schedule as well as NEIU’s academic calendar, and each login event was designated as occurring on a “class day” or “non-class day.” The median radial login phase was calculated for class days and non-class days for each individual per semester using the circular statistics toolbox [23] for Matlab. Averages of histograms of activity for each individual by gender and day type were calculated using means, as medians generated discontinuous outcomes that were not representative of daily distributions; histograms were normalized by gender, so that each gender had the same area under the curve for a given comparison, allowing comparison of distributions rather than absolute amount of activity. Pairwise comparisons between men and women utilized a paired *t* test. Correlations are Pearson’s correlations.

## Results

### Men are more variable than women as individuals and as a population

At NEIU, logging into the learning management system generates a user-specific timestamp. These data were de-identified, and entries were separated into those dated the same as a day on which that student had a registered class (“class day”) and all other days (“non-class day”). Each pair of class day and non-class day entry vectors was also associated with the gender of record at the university: men (*N* = 5887) or women (*N* = 7890). As we previously demonstrated [19], comparing the distribution of these login events across the day allows for the estimation of an individual’s average biological daily rhythms. For example, the distribution of these login events changes by season, age, and gender in ways expected of human circadian rhythms (e.g., the older the individual, the earlier in the day their logins are likely to begin). These natural sources of variation were all found to be significant on non-class days, whereas class days instead show spikes in login probability aligned to class onset times, which tended to mask natural sources of variance, like age, season, and gender.

**Fig. 1:**
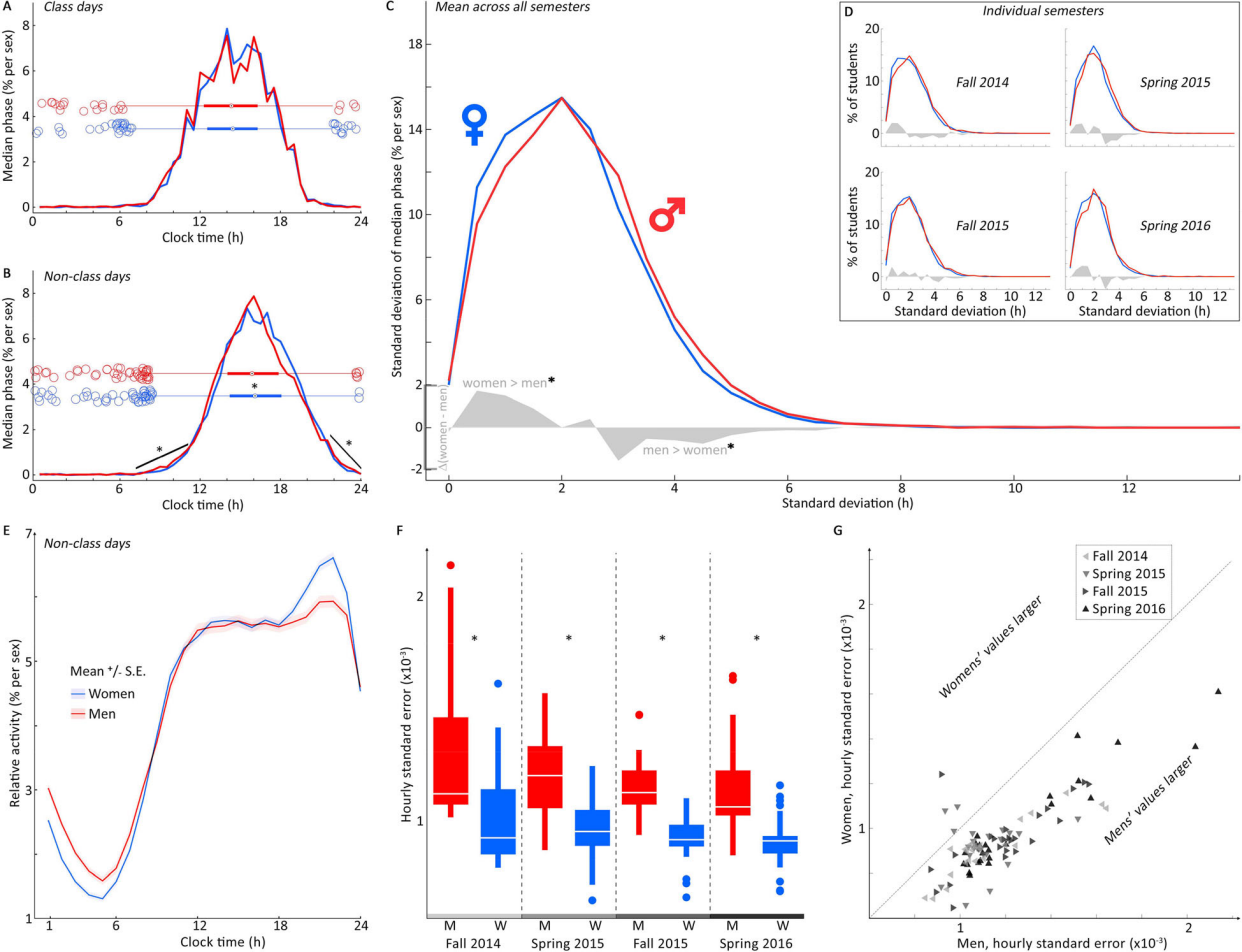
Men consistently exceed women in the variance of daily timing across semesters. Histograms with overlaid boxplots of median activity phase (men: red; women: blue; curves normalized within sex) on class days (**a**) and non-class days (**b**) find no difference on class days, but a significant delay of women relative to men on non-class days. Men are also shown to have significantly higher proportions at either extremely early or extremely late phases when compared to women (**b**, diagonal-indicated ranges). Histograms of standard deviation (SD) in the median activity phase across all semesters (**c**) reveals that low-SD individuals are significantly more likely to be women, while higher-SD individuals are more likely to be men (grey: difference of women and men). This pattern is consistent within each of the 4 semesters (**d**), not just across the average. The mean and standard error of the mean (SE) of activity by hour of the day across all semesters (**e**) reveals that men show less consolidated inactivity across individuals at night (midnight to 8am) than women, consistent with a wider range of chronotypes and individual variability observed. Conversely, women show a wider range of relative activity frequency as a population across an average non-class day (higher acrophase, lower bathyphase). Comparison of the hour-by-hour SEs of men and women’s activity (**f**) finds that men have higher average hourly SEs in all 4 semesters. Paired comparison of SE for the population mean of all individual’s means for each hour of the day across all four populations (24 h per average day/semester × 4 semesters = 96 comparisons) reveals that women’s SE exceeds men’s in only 4 of 96 comparisons. Shades of gray match from the bottom of **f** to triangles in **g**. *Significant; see the “Results” section for stats

To assess variability across individuals, we generated a histogram of the median phases of activity for each individual by gender and day type (histogram across individuals of median login activity by time of day calculated within individuals, normalized so each gender has the same area under the curve; Fig. 1a, b; boxplot overlays). Consistent with our previous observations, there was no detectable difference in phase histograms between the genders on class days (*χ*^2^ = 2.58, *p* = 0.11), but on non-class days, there was a small but significant delay in women relative to men (*χ*^2^ = 14.02, *p* = 0.002). Additionally, these histograms reveal that men composed disproportionately more of the extreme and outlier-phase individuals on non-class days (Fig. 1b, diagonal lines; paired *t* test, *p* = 0.0014), suggesting men as a population have more variance in chronotype (stereotyped daily phase) than women. Given the consistency of the day type effect in this finding and our previous work, we limited subsequent analyses to non-class days.

To assess variability within individuals, histograms of non-class day standard deviation (SD) were generated by gender (Fig. 1c, d). These revealed that on average, and consistently in all 4 available semesters, individuals with lower daily phase SD were more likely to be women, while those with higher SD were more likely to be men (Kruskal-Wallis of difference between genders by SD, lumped by SD (h) from 0:2h (to the shared peak) to 2.5:4.5h; *χ*^2^ = 12.94, *p* = 0.0003).

To assess variability within the day, the mean and standard error of the mean (SE) for activity in each hour of the day were calculated by gender (Fig. 1e, previously published [19]). Consistent with our findings in Fig. 1b, women showed a slight increase in evening activity, while men showed less concerted population-wide inactivity in the night. We compared the SE of each hour of the day as a population of SEs (Fig. 1f) and found that in every semester, men had a higher average hourly SE than women. We then directly compared SE hour by hour for each daily profile of each semester (24 h per average day/semester × 4 semesters = 96 comparisons), and in only 4 out of 96 paired comparisons did women have greater SE (Fig. 1g: points above the diagonal).

To summarize, men, not women, showed a higher likelihood of having extreme chronotypes, a higher likelihood of having higher individual SD of daily activity phase, and a higher SE in almost all hours of the day, and these patterns remained stable across all 4 semesters sampled. These findings do not agree with the belief that women are more variable than men and so ought to be considered potential statistical confounds when selecting subjects.

### Evidence for the importance of “sex as a biological variable”

**Fig. 2:**
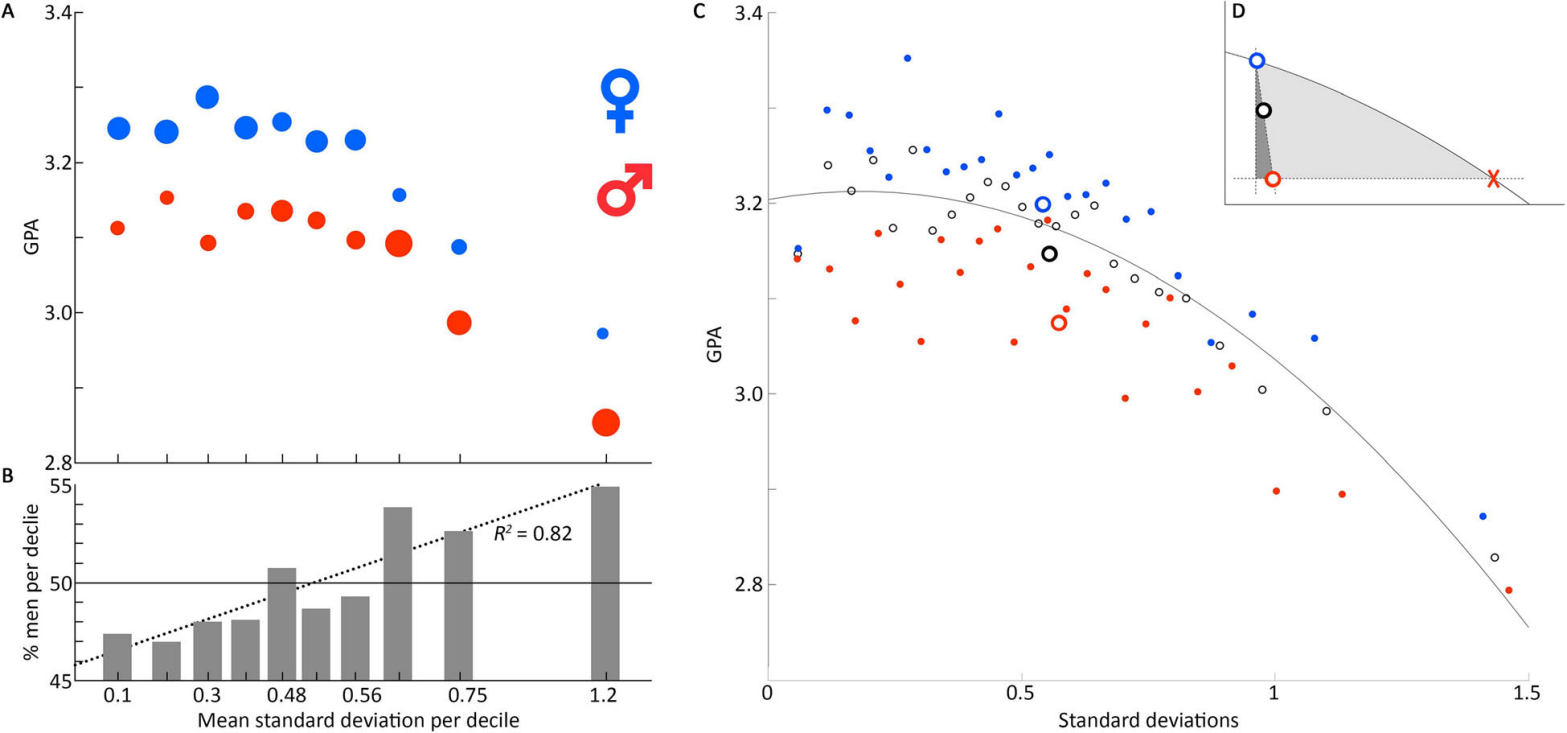
Effects of variance and gender on GPA are largely independent. Deciles of SD with equal populations (**a**) reveal men have a lower average GPA in all deciles. The size of each dot is proportional to the representation of that gender in each decile. Men show a significant increase in representation across deciles from the lowest to highest SD (**b**). Twenty-four divisions of the total population by SD amplitude (**c**, black rings) follow a polynomial decay of GPA with increasing SD. Separating these 24 by gender (red: men; blue: women) identifies a higher GPA for women in all fractions. Larger rings are population centroids. Comparing these centroids to the whole population polynomial fit curve (**d**) reveals that the GPA disadvantage of men corresponds to only 5.6% of the increase in SD necessary, were SD alone to account for the difference in GPA. SD is not the cause for the majority of the gender difference in GPA

The argument against using female subjects—that women are broadly and substantially more variable than men—is not supported by our initial findings. But a second argument contributing to a lack of female-specific research remains to be considered: that studies need not consider sex as a biological variable in analyses. In essence, the argument is that if females need to be included, all individuals of all sexes can be lumped in analyses, with the only impact being increased variance (implicitly due to inclusion of female subjects) [4, 11, 20]. To test this hypothesis that sex does not itself contribute to anything more than increased variance, we sorted the population by SD first, divided this into deciles, and then split each decile by gender. These deciles of SD-by-gender were then regressed against GPA (Fig. 2a, b). Contrary to the sex-only-affects-variance hypothesis, the two genders have distinct distributions: females have a higher average GPA in all deciles, and their proportional representation in each decile declines with increasing SD (Fig. 2a, b). Pearson’s correlation of the mean standard deviation per decile vs. percentage of men per decile is sufficient to quantify this trend (Fig. 2b; *r*^2^ = 0.82, *p* = 0.003).

Since a gender difference appears in both GPA and SD, and since there is a correlation across genders of increased SD to decreased GPA, it could still be argued that sex need not be considered in analyses, as differences in GPA might be proportional to differences in variance regardless of sex. To examine this possibility, we re-divided the entire population into 24 bins by amplitude of SD, with an equal population in each bin, and fit a 2nd-order polynomial to better capture the non-linear decline of GPA with increasing SD (Fig. 2c, black rings). We then binned each gender independently in the same way (Fig. 2c, blue: women, red: men) and compared each gender’s population to the whole population correlation trend, to assess whether the increase in GPA in women could be accounted for by the decrease in women’s SD (the centroids of each population are shown as larger, hollow circles, and are highlighted in the subpanel Fig. 2d). The difference between the men’s and women’s centroids is highlighted by the dashed line, and the darker-shaded region beneath the curve highlights the amount of the hypothetical horizontal traverse that is actually made by the male centroid. If gender differences in GPA were indeed because of SD alone, then the men’s and women’s centroids should fall roughly along the same trend line as determined by the whole population. Instead, the actual decrease of the men’s GPA is accompanied by only 5.6% of the expected increase in SD from the population-fit regression. Because the sex difference in SD is not proportional to the change in GPA with changing SD, we conclude that while gender differences in GPA are real, the difference in variance between genders only explains a minor amount of the overall difference in GPA between genders.

## Discussion

Our analysis here refutes the claim that ovarian cyclicity makes women more variable overall than men. We find the reverse to be true for daily timing choices, where men are more variable as a population (as in the range of medians across individuals) and within individuals (as in the variability of individual’s median daily phase). In previous work using continuous tracking in animal models, we found that males show higher overall daily variability and that this is in part due to their having a higher amplitude of within-a-day ultradian rhythms than females [16]. It is not possible to make that same comparison from the data analyzed here due to the lack of temporal measurement density, but the conclusions align, and so suggest possible future avenues of investigation in human sex and gender differences across timescales. It is worth noting that we do not know how many women in this sample are cycling, and so we assume some of the variances in the data from women come from ovarian cycles, but future studies on specifically cycling and non-cycling populations of women would clarify the extent to which ovarian cycles contribute to the overall variance seen in women.

Our work also identifies gender differences in academic performance beyond the differences caused by gender differences in variance (which turn out to account for a very small slice of the difference). This is consistent with previous findings [24], as is the finding that variance corresponds to decreased academic performance [25, 26]. It is interesting to note the polynomial relationship between individual variability and GPA. This relationship demonstrates that modest amounts of variability are not associated with substantial changes in GPA. Students might take heart that they need not slavishly adhere to schedules but might consider whether highly variable schedules could be impacting their performance (though we show no causal relationship here). Evidence exists for sex differences in tolerance to variability and schedule changes [27] but requires further attention.

It is widely appreciated that sex differences exist in human biology, in part due to the differences between the genetic landscape and physiology [21, 28, 29]. These variations lead to sex-specific differences in organs (e.g., kidney, liver, adipose tissue, and brain [30–32]) and in physiological responses, such as antioxidant defense [33, 34], immune function [35, 36], and stress [37, 38]. Any combination of these differences, and interactions with myriad social and cultural factors, could result in the differences in academic performance shown here and elsewhere [24]. Regardless of the specific mechanisms underlying the effects reported in this manuscript, it is clear that much work remains to be done before science and medicine can provide equitably for men and women (and the entire high-dimensional space not accurately reflected by that binary classification). For these future experiments to be successful, sex, and the way it is defined, will need to be considered as biological variables in analyses.

### Perspectives and significance

It is already national policy in the US that women should not be generally excluded as subjects in research. In spite of these policies, the belief that ovarian cycles make women more variable, and therefore experimental confounds, remains prevalent [11–15]. The prevalence of these beliefs has contributed to gender inequality, left female subjects substantially understudied, and put them at risk of negative health consequences that would be expected from this lack of data. Our findings add to a growing body of literature that variance from ovarian cycles should not be used to rationalize the exclusion of women from studies. Using a large, real-world data set, we find evidence that while gender differences in performance do exist, they are not driven by gender differences in variability over time. Lower performance and higher variability across time are both greater in men, not women, but the two effects are not strongly correlated. Our work therefore serves as a proof that women cannot be assumed to always be more variable than men. Given the breadth and impact of that historic assumption, proof that it must be tested in a case-by-case basis should give pause to those planning the current majority of experiments in which women are not included as subjects, or in which sex and/or gender is not included as a biological variable.

## Conclusions

While gender differences are real, women do not exceed men in overall variability in this data set, and so cannot generally be assumed to do so. What is more, gender differences in variability are not a key factor in the real gender differences observed here, either in the day-time activity phase or in GPA. We conclude that variability alone, whether dominated by men or women, should not be assumed to overwhelm experimental effects, but that the impact of sex/gender on experimental effects could be more easily assessed if experimenters routinely included sex/gender as biological variables when publishing.

## Supplementary Information

**Additional file 1.**

**Additional file 2.**

**Additional file 3.**

## Data Availability

All applicable code and data can be found in the supplemental materials.
